# A Scoping Review of Interventions for the Management of Nomophobia

**DOI:** 10.1007/s44192-026-00466-9

**Published:** 2026-04-30

**Authors:** Vardhini Krishnamurthy, Surekha Chukkali, Kasi Mayan Pavithrakshmi

**Affiliations:** School of Psychological Sciences, CHRIST (Deemed to be University, Dharmaram College Post, Hosur Road, Bengaluru, Karnataka 560029 India

**Keywords:** Nomophobia, Psychological interventions, Scoping review, Young adults

## Abstract

Nomophobia, or “no-mobile-phone-phobia”, refers to the anxiety and apprehension arisingfrom the inability to have constant access to mobile phones due to various reasons. In a worldwhere digital modes of communication and socialisation are becoming the norm and people are finding it increasingly difficult to survive without their phones, nomophobia is emerging as one severe consequence. This scoping review seeks to understand the breadth of the existing literature surrounding the interventions for nomophobia, along with understanding its formats, components, scales used, theoretical framework and outcomes. The search included studies published in English from 2008 to 2025 that assessed nomophobia as an outcome using standardised measures. A total of 4,828 records were identified, of which 12 articles met the inclusion criteria. The authors adopted the JBI framework to structure the scoping review. The included studies primarily focused on the young adult population. The interventions were grouped into mindfulness-based, psychoeducation, app-based, and multi-component, combining various techniques. Most of the interventions were administered in groups and in-person formats. The Nomophobia Questionnaire (NMQ) was the most commonly used tool. Results revealed the need for designing primary and secondary preventive measures and long-term follow-ups. The review highlights the critical need for interventions focused on nomophobia since it is still nascent. The interventions are focused on limited populations and are limited in scope. Given the public mental implications of this increasingly pressing concern, this study shows the urgent need for culturally adaptable interventions to foster healthier digital habits and overall well-being.

## Highlights

1. This study is one of the first scoping reviews to map the interventions targeting nomophobia.
2. Findings highlight the need for primary and secondary prevention of nomophobia, with a more rigorous methodological framework in the interventions.
3. Despite the increasing prevalence of nomophobia among young people, there are limited interventions addressing it.

## Introduction

Technology is advancing unprecedentedly, and smartphones have become integral to people’s lives. Smartphones assist people in all walks of life, including academics [1–2], social interaction [3–4], work [5], and entertainment [5–6]. Daily life entirely revolves around the availability of a smartphone. Although the convenience that comes with the presence of a smartphone can be very essential, it can give rise to the phenomenon of nomophobia, an abbreviation for “no-mobile-phone-phobia”. Nomophobia refers to the anxiety and apprehension arising from the inability to have constant access to mobile phones for various reasons [7]. Another related concept of mobile phone addiction often gets confused with nomophobia. However, mobile phone addiction is more about the excessive and uncontrolled use of mobile phones, leading to significant problems in psychological, physiological and social spheres of life [8]. This can be differentiated from nomophobia, which is more about the anxiety stemming from the unavailability of the smartphone rather than the addiction itself.

Reflecting on the multifaceted nature of nomophobia, researchers have categorised it in multiple ways. Some have conceptualised it as a behavioural addiction characterised by physical and psychological dependence [9]. At the same time, other researchers have understood it as a situational phobia similar to agoraphobia, given the manifestation of symptoms such as anxiety, palpitations, tachycardia, and respiratory distress in the absence of mobile phone access [10–12]. It has also been explained as a disorder of the contemporary digital era, resulting in significant anxiety, discomfort, and nervousness when individuals are disconnected from their mobile devices [13]. Although traditionally nomophobia has been defined as the fear and anxiety emerging from the inability to use the mobile phone [7], modern definitions today often term it as a situational modern-age phobia [14]. Individuals experiencing it usually have worries when they don’t have their phones around them, excessive control over them when they are with them, and always keep chargers with them. In a world where digital modes of communication and socialisation are integral to modern life, nomophobia is emerging as a pressing mental health concern. In light of this, mental health professionals and public health workers are increasingly proposing to include this as a disorder.

Theoretically, addictions have been primarily explained using the attachment theory [15], and nomophobia and excessive use of technology can also be described using the same framework. This perspective suggests that individuals form an emotional bond with their gadgets, similar to the one they create with their caregivers as a child. These individuals are called attachment targets. An attachment target cares for and protects the individual from any psychological danger [15–17]. Here, smartphones start functioning as attachment targets. Separation from the device causes them to develop anxiety similar to what they would experience as a young child being separated from the caregiver [18]. This attachment anxiety and insecurity have been found to predict the addictive and problematic use of smartphones [16–17, 19]. Examining nomophobia through attachment theory gives us insight into the emotional bonds individuals form with their devices and the resulting behavioural implications.

The attachment theory provides us with the lens to understand the emotional bond individuals form with their devices; however, the cognitive behavioural model can specifically explain the thoughts, emotions, and behaviours associated with it [20]. An individual may believe that if they don’t have their phone with them, they will miss out on something important, hence leading to anxiety and maladaptive behaviours like constantly carrying their phone around, checking it and keeping it fully charged. These behaviours only provide temporary relief, but over time, they become a cycle that is difficult to break. The CBM approach complements the attachment theory by giving us a framework for managing nomophobia, which is essential for this study.

Nomophobia is gradually becoming increasingly prevalent in most parts of the world, among various demographic groups. Based on the findings of a systematic review conducted among university students, Indonesia had a prevalence rate of 71%, while Germany had the lowest rate of 3%. Data drawn from a synthesis of research across 18 countries, involving a combined sample of 36,656 individuals, indicate that approximately 21% of participants experience severe symptoms of nomophobia, 56% report moderate symptoms, and 26% exhibit mild symptoms [21]. Duration of smartphone ownership is associated with nomophobia, where people who have owned smartphones for over 3 years [22] and used them for more hours a day [22–25] have higher levels of nomophobia. Regarding age, research has shown that individuals under the age of 35 are known to exhibit higher levels of nomophobia compared to older individuals [26–29]. Among these, the 18 to 24-year-old age group is estimated to have 77% of the population affected by nomophobia. This is attributed to their familiarity with technology and fewer family constraints than older people [30].

Individuals affected by nomophobia often display distinct and recognisable behavioural symptoms. These include spending considerable time with their phone, carrying chargers due to the lack of charge, and fearing misplacing the phone [13]. This leads to avoiding places that do not allow phones, a constant urge to check if they have received any calls or messages, and a compulsion to always keep their phones with them, even while asleep. There is also a preference to use phones for social interaction compared to face-to-face interaction [7, 31–32] and much money is spent on phones and their maintenance [13]. Individuals with nomophobia often experience significant anxiety and stress when they are unable to access their mobile phones. This anxiety can be severe and is a primary predictor of nomophobia [21, 33]. Physical symptoms are also associated with the condition, such as musculoskeletal problems emerging due to excessive phone usage and insomnia [33–34]. Individuals with nomophobia also end up having maladaptive coping strategies like self-distraction, denial, and venting when faced with stress [11].

Nomophobia’s aetiology is associated with various physical, demographic and psychological factors, along with demographic factors like age, gender and socio-economic factors. Studies have shown evidence of the association of nomophobia with fear of missing out or FoMO [35], personality factors like extraversion, agreeableness, and conscientiousness and anxiety, depression and stress [10, 28, 36]. High levels of stress and loneliness also may cause individuals to spend more time on their phones, and the fear of being disconnected from their phones, especially when feeling lonely [37]. Having lower levels of emotional intelligence and poor problem-solving skills has also been associated with having high levels of nomophobia [38].

Apart from individual predictors, empirical research has also identified structural components of nomophobia based on structural modelling studies. The foundational nomophobia questionnaire looks at the concept as being multidimensional, including fear of inability to communicate, losing connection, inability to access information, and issues with lack of convenience [7]. Other studies also describe the underlying structure of nomophobia as including distress when losing connection, reduced productivity due to phone use, and the tendency to keep the internet on and use the phone even after being exhausted. Subsequent studies have sought to adapt this questionnaire into other languages to enhance cultural validity and to develop new scales for greater robustness. For example, a structural equation modelling study showed that netlessphobia and age contributed to the nomophobia model, with greater importance than internet dependence and other developmental variables [39] A better understanding of the factors can help design theoretically better-informed interventions, as it demonstrates that nomophobia is a complex structure combining cognitive, emotional, and behavioural elements.

Nomophobia is not just an individual condition but also a collective one. It has an impact on the mental health of the individuals who are facing it because it leads to an increase in the levels of anxiety, depression and stress [40]. However, the increasing reliance on digital communication has significantly affected the quality of social interactions. The shift from in-person to online modes of communication leads to a gradual decline in individuals’ comfort and proficiency with in-person social engagement. As a result, when offline interaction becomes necessary, individuals may experience heightened levels of social anxiety [41–42]. There is also a significant impact that nomophobia has on work and academics. Constantly getting distracted by one’s phone can make one less productive at work, and individuals may experience higher levels of work stress [27]. Students are finding it hard to focus and sustain attention on academics due to constant notifications and distractions. This leads to higher stress at school and college and poorer results [43].

Various tools and questionnaires have been developed to assess nomophobia and measure its severity and impact. However, the most popular one is the Nomophobia Questionnaire [7]. It is a 20-item questionnaire, and all items are scored on a 7-point Likert scale, with one being “Strongly Disagree” and seven being “Strongly Agree”. Other than this, mobile phone addiction scales and social media usage questionnaires are used interchangeably with the Nomophobia Questionnaire; however, they do not measure the exact dimensions required to calculate this condition. The Indian Scale of Assessment of Nomophobia [44] has just been developed and validated, which provides a good alternative for the Nomophobia Questionnaire.

Though research has been done in the area of nomophobia and smartphone use, there have been comparatively fewer articles that have spoken about interventions. Studies have primarily focused on risk factors and characteristic features. However, awareness about the condition remains very low, and intervention studies in this area are scarce. Since very few studies have been conducted to understand this condition, there is a significant need to understand the depth and efficacy of the existing interventions to tackle nomophobia [27]. Thus, employing a scoping rather than a systematic review seemed more applicable, which would help adopt a broader perspective and gather evidence from various aspects of the literature [45]. A scoping review approach was chosen to explore peer-reviewed literature on interventions for nomophobia. This method helped understand the depth of existing research and summarise key findings to guide future studies. It is essential to know that this review is confined to interventions aimed at nomophobia rather than mobile phone addiction or dependence. This review seeks to highlight strategies to deal with the anxiety of not having smartphone access rather than the addiction, since digital addiction has been studied extensively in the past. This further suggests that interventions targeting attachment anxiety may help mitigate the severity of nomophobia.

## Materials and methods

This study aimed to understand the existing interventions for managing nomophobia, the populations they serve, and their outcomes and limitations. This scoping review was conducted according to the methodological guidelines provided by the Joanna Briggs Institute for scoping reviews [46–47]. The search terms were identified from the review questions and used to locate available literature. The selected studies were then extracted and thoroughly reviewed, and the results have been reported.

### Review questions

What is known from the existing literature about the different interventions developed to address nomophobia?

What are the core components of nomophobia intervention programs, and in what ways have the included studies evaluated their measurement and reported on their effectiveness?

### Aim

To map the existing evidence of interventions for nomophobia, focusing on summarising the intervention content to inform future research in the field.

###  Search strategy

The search strategy aimed to locate published and unpublished studies such as journal articles, case reports, dissertations, newspaper reports, conference proceedings and web pages. The search was conducted in three stages. First, an initial search of Scopus was undertaken to identify articles on the topic. The text words contained in the titles and abstracts of the articles were then used to create a complete search string that can be used in all other databases. After that, a sensitive search strategy was conducted using Scopus, PubMed, APAPsycNET, EBSCOHost, JStor, Cochrane Library, Science Direct, Google Scholar and ProQuest from March to April 2025. Search terms were: ((“Nomophobia“[Title/Abstract]) AND (“intervention” OR “treatment” OR “therapy” OR “CBT” OR “mindfulness” OR “relaxation training” OR “group therapy” OR “self-control” OR “Digital Detox” OR “Psychoeducation”)) AND (english[lang]) AND (2008:2025[dp]). In some searches, due to the emergence of various irrelevant results, two keywords, e.g. Nomophobia AND Intervention, were applied in the search criteria post, and the following command of Nomophobia AND Therapy was searched. Finally, a snowballing search was conducted, and the reference lists of all included studies were searched for any further sources that would fit the criteria.

Reviews that studied the effect of any intervention with nomophobia as the outcome variable were included in the study. The primary inclusion criteria followed for the studies were: (1) the studies published in English from January 2008 to March 2025 since the term was initially coined in the year 2008 (2) nomophobia was assessed as one of the outcomes in the intervention (either primary or secondary) and the (3) studies that used standardised measures to assess the levels of nomophobia. The interventions comprised single- or multi-component formats and were delivered individually or in group settings. To review, the requirements of a control group and participant randomisation were not mentioned in the inclusion criteria of the studies. Studies that used terminologies other than nomophobia, like smartphone dependence, problematic use, etc., were excluded to avoid studies that may be directly applicable to the objective. Books and book chapters were excluded due to time constraints. No limitations were placed on the population included in the study. Review articles were not included. However, the references mentioned in them were used for snowballing articles.

For the present study, nomophobia or “no-mobile-phone phobia” has been defined as the anxiety or apprehension arising from the inability to use mobile phones due to various circumstances. It encompasses being unable to communicate, access information via the phone, losing connectedness and giving up the convenience of having the phone.

### Selection of studies

The process of selecting the studies had four significant steps. First, an initial pool of studies was identified from the databases in Table 1. Once completed, the chosen pool was screened for title and abstract relevance. The full texts of the selected records were then assessed for eligibility based on the inclusion criteria. Snowballing was also done using the reference lists of the selected articles to locate studies. Records that met the inclusion criteria in this final step were included in the review. The retrieved documents were entered into the Rayyan [48] software to screen for duplicates. Two researchers performed the scientific selection independently, and minor disagreements were resolved through discussion and consensus.

**Table 1:** Search strategy

Database	Search terms used
Scopus	((“Nomophobia“[Title/Abstract]) AND (“intervention” OR “treatment” OR “therapy” OR “CBT” OR “mindfulness” OR “relaxation training” OR “group therapy” OR “self-control” OR “Digital Detox” OR “Psychoeducation”)) AND (english[lang]) AND (2008:2025[dp])
PubMed	((“Nomophobia“[Title/Abstract]) AND (“intervention” OR “treatment” OR “therapy” OR “CBT” OR “mindfulness” OR “relaxation training” OR “group therapy” OR “self-control” OR “Digital Detox” OR “Psychoeducation”)) AND (english[lang]) AND (2008:2025[dp])
APA PsycNET	((“Nomophobia“[Title/Abstract]) AND (“intervention” OR “treatment” OR “therapy” OR “CBT” OR “mindfulness” OR “relaxation training” OR “group therapy” OR “self-control” OR “Digital Detox” OR “Psychoeducation”)) AND (english[lang]) AND (2008:2025[dp])
EBSCOHost	((“Nomophobia“[Title/Abstract]) AND (“intervention” OR “treatment” OR “therapy” OR “CBT” OR “mindfulness” OR “relaxation training” OR “group therapy” OR “self-control” OR “Digital Detox” OR “Psychoeducation”)) AND (english[lang]) AND (2008:2025[dp])
JStor	((“Nomophobia“[Title/Abstract]) AND (“intervention” OR “treatment” OR “therapy” OR “CBT” OR “mindfulness” OR “relaxation training” OR “group therapy” OR “self-control” OR “Digital Detox” OR “Psychoeducation”)) AND (english[lang]) AND (2008:2025[dp])
Cochrane Library	((“Nomophobia“[Title/Abstract]) AND (“intervention” OR “treatment” OR “therapy” OR “CBT” OR “mindfulness” OR “relaxation training” OR “group therapy” OR “self-control” OR “Digital Detox” OR “Psychoeducation”)) AND (english[lang]) AND (2008:2025[dp])
ScienceDirect	((“Nomophobia“[Title/Abstract]) AND (“intervention” OR “treatment” OR “therapy” OR “CBT” OR “mindfulness” OR “relaxation training” OR “group therapy” OR “self-control” OR “Digital Detox” OR “Psychoeducation”)) AND (english[lang]) AND (2008:2025[dp])
Google Scholar	((“Nomophobia“[Title/Abstract]) AND (“intervention” OR “treatment” OR “therapy” OR “CBT” OR “mindfulness” OR “relaxation training” OR “group therapy” OR “self-control” OR “Digital Detox” OR “Psychoeducation”)) AND (english[lang]) AND (2008:2025[dp])
ProQuest	((“Nomophobia“[Title/Abstract]) AND (“intervention” OR “treatment” OR “therapy” OR “CBT” OR “mindfulness” OR “relaxation training” OR “group therapy” OR “self-control” OR “Digital Detox” OR “Psychoeducation”)) AND (english[lang]) AND (2008:2025[dp])
Snowballing	Manual reference list search using citation chaining from included studies to locate relevant literature on interventions for nomophobia

### Data extraction

**Fig. 1:**
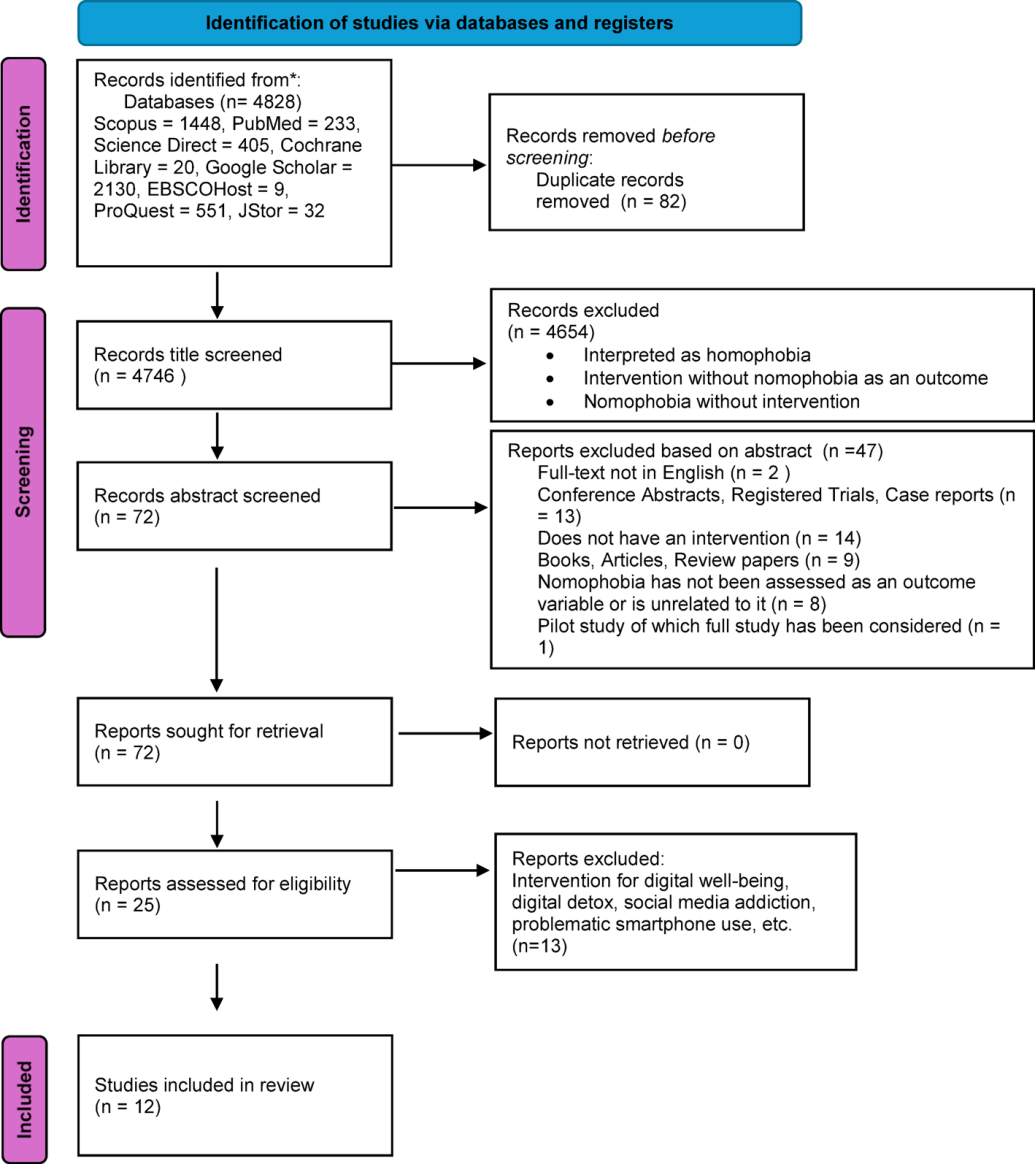
PRISMA flow chart of included studies

A PRISMA flow diagram delineates the article selection process (Fig. 1). The data were also charted on the authors’ Microsoft Excel data extraction sheet. This included manuscript characteristics like author, year, country and title, as well as study characteristics like aim, population, study design, tools used, key findings, limitations and future research, along with intervention characteristics like type of intervention, theoretical basis of the intervention, mode of delivery and duration of the intervention, individual/group, presence of a control group, and single or multi-component. Any uncertainties related to data extraction were discussed with the research team.

Four thousand eight hundred twenty-eight articles were initially retrieved from the databases listed in Table 1. Among these, 82 articles were deleted after deduplication. Titles were then screened, leading to the exclusion of 4,654 papers. Abstract screening was conducted for the remaining 72 articles. Of these, 47 were excluded, leaving 25 articles to be included in the full-text review, as shown in Table 2. Of the 47 excluded articles, their full texts were available only in foreign languages. Eight were unrelated to nomophobia or did not assess nomophobia as an outcome variable. Fourteen did not include an intervention as part of the study. Thirteen were conference abstracts, registered trials, or case reports without published research, and nine were books, general articles, or review papers. One was a pilot study; however, the complete version has been considered part of this paper. The remaining 25 papers were included in the full-text screening, of which 13 were excluded as they focused on interventions for problematic smartphone use, digital detox, social media addiction, digital well-being, etc., and not specifically on nomophobia. Finally, 12 studies were included in the review.

**Table 2:** Key features of included studies

Author/Year/Country	Aim/Purpose	Population and size	Study design	Intervention type	Duration of intervention	Key findings
Throuvala et al., [51], UK	To evaluate the effectiveness of online app-based interventions on various aspects of mobile phone usage	University students (*N* = 252) before dropouts	RCT (pre-post with control group)	Headspace App (based on brief mindfulness sessions), Anti-Social App (Social media and smartphone use abstinence option), & Pacifica App	10 days	Significant reduction in smartphone distraction, mindful attention, emotional awareness, stress, anxiety, self-efficacy, deficient self-regulation, FoMO, problematic use, impulsivity; no significant change in levels of nomophobia, social media use
Safaria et al., [50], Indonesia	To explore the effect of a spiritually oriented mindfulness program on nomophobia	Muslim College students (*N* = 66), moderate to high level of nomophobia	RCT (pre-post with control group)	Spiritual Psychology & Mindfulness-Based Intervention	15 sessions in 7 days, 1.5 h/per session	Reduced nomophobia in both groups, with a higher reduction in the experimental group. Follow-up: positive effects of the intervention persisted in the experimental group.
Maghfiroh et al., [55], Indonesia	To examine the effectiveness of mindfulness training in reducing nomophobia among Gen Z remote workers and evaluate differences in nomophobia based on Big Five Personality Traits.	Employees of a media company (*n* = 10) aged 20–27 years, with moderate to severe levels of nomophobia.	Quasi-experimental with a one-group pretest-posttest design.	Mindfulness training	4 sessions, 14 days, 1.5 h/ per session	Significant reduction in nomophobia levels after mindfulness training. Based on personality, extraverts experienced the highest reduction in nomophobia after the intervention
Singh & Sharma, [58], India	To provide a comprehensive overview of the potential effects of guided imagery, sports activity and composite training in the management of nomophobia.	200 college students screened, 80 selected (18–24 yrs)	Experimental pretest-posttest control group design (4 groups)	Guided Imagery, Sports & Composite Training (Guided Imagery + Sports)	Not specified	All reduced nomophobia; sports are most effective
Prasyatiani et al., [60], Turkey	To introduce a behavioural program - Seven Magic Days (SMD) to address nomophobia	Not specified	Descriptive conceptual framework	CBT, group therapy, detox, and games are all-inclusive in the Seven Magic Days	7 days	Proposed model
Devi & Masih, [54], India	To test whether psychoeducation can reduce nomophobia in rural adults	Adults aged 20–49 (*n* = 600)	Quasi-experimental with a one-group pretest-posttest design.	Psychoeducation	Not specified	Nomophobia significantly reduced
Fernandez-Crespo et al., [49], Spain	To evaluate the impact of a Neurofeedback-Assisted Mindfulness Training Program (NAMTP) on disorders associated with problematic use of mobile phones.	Young adults (18–35 years) (*N* = 40)	Randomised controlled clinical trial with two parallel groups	Neurofeedback-Assisted Mindfulness Training Program (NAMTP) + Educational Workshop, Control group - Only Educational Workshop	25 sessions (2–3/week) during 3 months	Proposed model
Ortega-Barón et al., [57], Spain	Evaluate the effectiveness of the Safety.net program designed to prevent relational and dysfunctional internet risks in a normalised context.	Adolescents (11–14 years) (*N* = 726)	Pre/post-test repeated-measures design with an intervention group (*n* = 450) and a control group (*n* = 276)	Multi-risk internet prevention program (Safety.net)	16 sessions (1 h/session)	The intervention group showed significant improvements compared to the control group in peer cybervictimization, cyber dating victimisation, sexual solicitation/interaction with adults, problematic internet use, and most improvement in reducing nomophobia.
Ali et al., [53], Egypt	This study aimed to evaluate the efficacy of a psycho-educational program on nomophobia and social media addiction among undergraduate nursing students.	Undergraduate Nursing Students (*N* = 260)	Quasi-experimental (pre-post one-group) design	Psychoeducational program	1 h/weekly, six sessions	Post-intervention, high and moderate levels of nomophobia shifted to moderate, signifying substantial statistical disparities in overall nomophobia scores.
Bakariwie et al., [59], Ghana	This study investigated nomophobia prevalence among students of a senior high school in Ghana, designed and implemented interventions to ameliorate the condition and assessed the impact of the interventions.	Senior high school students (*N* = 70)	Action research design	Strength-based counselling, co-curricular activities, and blended learning	Not specified	Pre-intervention analysis revealed alarming levels of nomophobia, with 73% and 27% reportedly suffering severe and moderate levels of nomophobia, respectively. Post-intervention, a reduction in nomophobia levels was observed, with 79% and 21% reporting moderate and mild levels of nomophobia, respectively. No one was cured entirely, but neither was there anyone with severe levels.
Nasab et al., [56], Iran	To evaluate the effectiveness of a nomophobia therapy package on self-esteem and nomophobia symptoms among high school students.	30 high school students (15 experimental, 15 control)	Quasi-experimental (pretest-posttest-follow-up with control group)	Nomophobia Therapy Package	8 weekly sessions (75 min each) + 2-month follow-up	Significant improvement in self-esteem and reduction in nomophobia symptoms, effects sustained at follow-up
Torpil & Pekçetin, [52], Turkey	To compare the effectiveness of client-centred and time management interventions on nomophobia, time management, and occupational performance in university students with severe nomophobia.	46 university students with severe nomophobia (23 in each group)	Single-blind randomised controlled trial	Client-Centred Intervention and Time Management Intervention	10 sessions (45 min each), two sessions/week for 5 weeks	Both interventions significantly improved nomophobia, time management, and occupational performance; the client-centred intervention had more potent effects.

## Results

### Characteristics of included studies

Of the included studies, four were randomised controlled trials [49–52], four quasi-experimental studies [53–56], two experimental studies [57–58], one action research [59] study, and one descriptive conceptual research framework [60].

The studies were conducted across developed, developing and underdeveloped countries. Specifically, two studies were conducted in Turkey [52, 60], Spain [49, 57], Indonesia [50, 55], and India [54, 58], with one study in Ghana [59], the UK [51], Egypt [53] and Iran [54]. In terms of year of publication, the oldest study was published in 2017, and the most recent one in 2025 [55]. The data shows that nomophobia has been comparatively more recent since the advent of smartphones, and the coining of the term has also been recent. The articles looked at different populations, though most focused on young individuals. Some studies examined school-aged populations, including high school students [56, *n* = 30] and senior high school students [59, *n* = 70], covering all age groups, with one specifically looking at adolescents [57, *n* = 726].

Apart from school students, majority of the studies focused on university students, including nursing undergraduate students [53, *n* = 260], and university students [51, *n* = 252; 50, *n* = 66; 52, *n* = 46) A few studies also included broader adult populations ranging from 18 to 49 years of age [54–55, 49, 58]. However, the higher number was predominantly in adolescents and young adults.

### Nomophobia intervention strategies and their components

The interventions included in this review varied widely in terms of their design, theoretical foundation, and delivery. The articles were analysed, and common patterns were looked for to understand and categorise the interventions used in this study. We have reviewed the interventions based on the core mechanisms they have adopted, and we have clustered them as cognition, emotion, behavioural and eclectic interventions. This model helps understand that each intervention either looked at changing maladaptive cognitions, better regulation of emotions, altering problematic behaviours, or combining these to reduce or manage levels of nomophobia.

#### Cognitive interventions (*n* = 4)

 These interventions focused on changing maladaptive thoughts and beliefs or instilling thoughts that focus on helping people understand the concept of nomophobia and how it may impact them. Two studies employed psychoeducation-based interventions [53–54]. These sessions were conducted using video lectures and interactive discussions in a classroom setting, which helped explain to the participants what nomophobia is and how it may affect them. Considering that nomophobia is a new phenomenon, such interventions are beneficial in educating individuals on unfamiliar topics and helping bring their attention to this problem they may fall prey to [61]. It will help challenge the irrational beliefs that individuals have surrounding the phenomenon. Working on the thought aspect would be a primary step in changing emotions and behaviour. Similarly, “Seven Magic Days” [60] used CBT alongside motivational interviewing to promote self-regulation after a one-week phone-free period. It was given along with support group therapy, which helped me appreciate the presence of people around and gain motivation from others who are going through similar negative emotions. The Safety.net program [57] was another interesting intervention that provided a multi-risk internet prevention program to address all major cyber issues within the same module. The program sought to educate individuals on the risks posed by technology and how to change their attitudes and behaviours to help combat these risks. Programs were teaching them basic digital skills like netiquette and digital privacy. They were also educated on relational risks, which included cyberbullying, sexting and online grooming. The next module involved education on dysfunctional risks like online gambling, nomophobia and FoMO and lastly, there was a module on changing one’s attitudes and cognitions.

#### Emotion interventions (*n* = 4)

 The emotion-based interventions attempted to focus on the anxiety, stress and dysregulation that would be experienced when the phone is taken away or there is a chance of having to be without one’s phone. The primary mode of emotion-focused interventions was using mindfulness techniques. These interventions shared a common goal of making people aware of the present moment and developing an overall awareness of themselves, including their physical, emotional and cognitive state. Across the studies, mindfulness was used to help individuals regulate anxiety, improve emotional clarity, and build resilience in the absence of their mobile phones. At the same time, these shared principles formed the foundation of all three interventions, each uniquely incorporating mindfulness. One study [50] tried to combine Islamic teachings and mindfulness concepts, terming it “spiritual mindfulness”. As a part of the intervention, each day focused on bringing awareness of a particular body part along with the Dhikr repetition, which helped align the individual’s spirituality and present moment awareness, both of which help cope with stress. A combination of physical sensations, thoughts, emotions, and perceptions provided a holistic understanding of the person’s current state of being. Another study [49] combined mindfulness with neurofeedback mechanisms.

Whereas traditional neurofeedback relies solely on EEG and brain wave activity to provide feedback on cortical functioning, this intervention enhances the approach by integrating calm mental states, focused attention, and reinforcing them through rewards. Doing so offers users neurophysiological feedback and a clear neurological explanation of how mindfulness contributes to reduced anxiety, highlighting the experiential and biological mechanisms underlying its effectiveness. The third study [55] was rooted in traditional mindfulness principles, each lasting 90 min and four sessions over two weeks. Together, these interventions demonstrate mindfulness’s flexibility and effectiveness in managing nomophobia. Mobile app-based interventions were also used to track emotions and help in regulation. Positive psychology-based components were also a central feature in interventions such as nomophobia therapy [56], which included self-esteem, self-acceptance, assertiveness, interpersonal communication, health conversation, self-efficacy and other positive psychology-based concepts to help overcome isolation, anxiety, combat negative thoughts and destructive beliefs. The researchers believed that all these would together help reduce levels of nomophobia, which is why it was termed nomophobia therapy.

#### Behavioural interventions (*n* = 1)

 Behaviour-based interventions focus on equipping clients with techniques to change their behaviours related to phone usage or nomophobic tendencies. Behaviour change will directly impact improvements in mood, stress levels, and changes in thoughts. Two intervention approaches tested in a study were grounded in occupational therapy’s time management principles and client-centred therapy [52]. These approaches helped modify the behaviour surrounding mobile phone usage, along with a small cognitive component to sustain the behaviour change. The first intervention tried to help individuals form goals, determine the pros and cons of situations, make plans and take action, which helped reduce anxiety and promote holistic development. The second one catered towards time management and building a schedule where time was allocated for self-care, sleep, leisure, and all day-to-day tasks. Both aimed to improve the management of Activities of Daily Living (ADLs) and ultimately reduce symptoms of nomophobia [52]. Helping improve ADLs would ultimately help modify mobile-phone-related maladaptive behaviours.

#### Eclectic interventions (*n* = 3)

 Some interventions combined aspects of more than one particular modality, such as a mix of cognition, emotion and behaviour, to target more than one component at a time. Mobile app-based interventions have proven to improve self-awareness and regulation by tracking an individual’s behaviour on their phone and the time spent online. Building apps to help prevent distraction and regulate activity is a recent concept that is gradually taking precedence in the literature. This study [51] assessed three different apps: the first focused on mindfulness and mood tracking to improve focus, and the second focused on self-monitoring and emotion regulation. In contrast, the third one was focused entirely on mood tracking. Combining these apps helped focus on the components of emotions through mindfulness, mood tracking, and behaviour by ensuring self-monitoring of the duration spent on the phone.

Another intervention introduced a composite training program combining guided imagery, sports and composite training, which is a combination of the two to address the negative thoughts, emotional dysregulation, and physical symptoms associated with nomophobia [58]. This helped target cognitions, emotions and physical symptoms through one therapy package.

A related intervention [59] incorporated strength-based counselling, participation in co-curricular activities, and blended learning (online and in-person) to reduce nomophobia. It tried to use all three of these to reduce the students’ nomophobia levels. The first is to reduce stress and cope mentally, the second is to make the students more physically active, and the last is to equip them with skills to use their digital devices more cautiously and mindfully.

### Features of nomophobia intervention

 Regarding the modality used for the sessions, 83% of the studies were delivered in person, while only 17%, or two studies, had an online mode of delivery. The studies conducted in person were in classrooms, therapy rooms, or camp settings [49–50, 55, 57–58, 60]. Two of the studies used an online mode of delivery—Throuvala et al. [51] used a mobile app, while Ali [53] delivered psychoeducation sessions through Zoom, PowerPoint, and pamphlets. One study adopted a unique hybrid format that combined in-person sessions, co-curricular activities, and online modules [59].

All interventions focused on reducing levels of nomophobia. Some studies focused solely on nomophobia [50, 52, 54, 58, 59]. In contrast, others addressed nomophobia along with variables such as problematic internet use, cyber victimisation, self-esteem, social media addiction, personality traits, anxiety, psychological distress, smartphone distraction, and online vigilance [49, 51, 53, 55–57].

The duration of the intervention programs varied considerably, depending on the type of intervention. Some were short-term and intensive, especially those involving free app usage, ranging from 7 to 10 days [51, 60]. Others involved standard 90-minute sessions delivered over one to two weeks [50, 55]. Longer interventions ranged from 6 sessions [53] to 16 sessions [57] to 25 sessions delivered over three months [49].

Considering the format of delivery, since nomophobia is often conceptualised as a part of mobile phone addiction, many interventions followed a group format [49–50, 53, 55, 57–58]. A reason for this could be the belief that group settings enhance motivation, accountability, and scalability. A few interventions were delivered individually [51–52], while some combined individual and group sessions to maximise output [59–60].

### Scales used in the studies

 The studies used various psychometric tools to assess the different variables they considered. However, while considering nomophobia, most articles have used the Nomophobia Questionnaire (NMQ) developed by Yildirim & Correia [7]. The questionnaire is a 20-item scale with a 7-point Likert scale ranging from “strongly disagree” to “strongly agree”. It categorises the individuals based on their levels of nomophobia: absent, mild, moderate and severe nomophobia. Since nomophobia is a relatively new phenomenon, this is the only reliable, validated scale available in the last decade and has been used widely across most studies, including the ones in this review [7, 49–53, 55–56, 58].

However, for greater cultural validity and adaptability, two Indian studies created their versions of a nomophobia questionnaire [54, 58]. Most studies were not only concerned with nomophobia but also studied other variables. These studies used various scales along with the NMQ, like those for personality, positive psychology variables and other internet-based addictions and difficulties.

### Outcomes of the studies

 After assessing all the interventions used in these studies, it is evident that most have shown positive results in reducing levels of nomophobia. Upon reviewing the interventions, it was observed that the emotion interventions, based explicitly on mindfulness, were the most common [49–50, 55, 62]. Cognitive interventions like those centred around psychoeducation [53–54] and the safety program [57] also showed a significant decrease in the levels of nomophobia on the post-assessment. The behaviour intervention was significantly effective in reducing levels of nomophobia. The study, which compared two programs, indicated that both effectively reduced levels of nomophobia. However, the client-centred therapy was more effective than the time management intervention [52]. However, among the multi-component interventions, the technology-regulated interventions [51] did not show a reduction in the levels of nomophobia. The program, which compared guided imagery, sports, and composite training sports activities [58], emerged as particularly practical.

## Discussion

This review synthesises studies related to interventions developed to address nomophobia. Since smartphones have become an essential part of our lives, nomophobia is emerging as a consequence of this addiction [63]. Given the significant impact of smartphone dependence on personality development and mental, physical, educational, and social well-being, particularly within adolescent populations, understanding effective interventions is essential [64–65]. Although numerous studies have examined the prevalence, risk factors and correlates of nomophobia, very few have proposed or evaluated evidence-based approaches to mitigate it. This scoping review highlights the limited research available on interventions for nomophobia. The interventions have been categorised based on the cognitive behaviour model [20] to help provide a framework for organising the results. Consequently, this review aims to consolidate existing intervention strategies, highlighting their efficacy and identifying gaps for future research directions. This scoping review addresses this lacuna by critically appraising existing intervention studies, shedding light on their methodologies, outcomes, and the extent to which they align with the complex nature of nomophobia as a psychological construct [25].

### Participant demographics

Most interventions targeted adolescents and university students, confirming this demographic as highly susceptible. This increased vulnerability may be because today’s youth are tech-savvy and are brought up in a digital world. Smartphones are a part of their development process from an early age, be it for learning or recreation, resulting in a high level of dependence on the gadgets for everyday functioning. This finding is supported by previous research by Joe & Linson [66] and Vagka et al. [67], highlighting the need for nomophobia interventions for youth. However, nomophobia is now gradually emerging as a threat even for older adults, resulting in cognitive decline, memory loss and disorientation [68]. Therefore, it becomes necessary to consider suitable nomophobia interventions for older adults.

Nomophobia is growing into a global issue. The growing prevalence of nomophobia among adolescents underscores an urgent need for structured programs and interventions, but research attempts seen so far have focused less on management. The few interventions reported were also limited to specific regions. Most existing studies focused on understanding the prevalence and the associated risk factors. It was interesting to note that the present scoping review had studies from Europe, Asia and Africa reporting a significant increase in nomophobia, particularly in countries like Ghana [59], Indonesia [21] and Saudi Arabia [69].

Contributions of studies [52, 60, 59, 52, 57] from Turkey, Ghana, and Indonesia are pioneering in nomophobia research as they particularly contribute to measurement and cultural specificity informing evidence-based intervention research. Research from India similarly contributes to the field by offering both intervention-based findings [54, 58] and scale development [44] efforts that enhance the cultural adaptability and contextual relevance of nomophobia assessment and intervention.

### Delivery formats of the interventions

The format of intervention delivery is one of the essential features contributing to the efficacy of interventions. In this review, it was seen that the studies mainly used an in-person delivery format, though there were attempts at adopting the other two delivery modes as well. Only two studies employed online modality [51, 53], and one adopted a hybrid approach [59], combining online and offline strategies. The preference for in-person delivery over online appears beneficial since nomophobia itself results from an excessive dependence on gadgets and is supported in research studies by Alrobai et al. [70] and Jones et al. [71]. They explain the risk involved in digital interventions, wherein we reinforce the behaviours the intervention seeks to change. In-person interventions can help the person detach from the device physically and mentally, reducing the overreliance. It also supports healthier digital habits. The finding can also be understood from the lens of the behavioural addiction theory [72], which explains how the online or technology-related interventions may trigger the same reward pathways that maintain nomophobic tendencies. According to this theory, behavioural addiction is similar to substance addiction and shares the same features. Smartphone notifications provide constant rewards and access to information, activating the dopamine reward pathway and reinforcing repeated phone usage. These interventions, although well-intentioned, often require individuals to remain engaged with their devices, reinforcing patterns of compulsive checking and dependence. Repeated exposure to the phone can maintain or even heighten anticipatory anxiety when disconnected, as in the case of nomophobia.

### Need for primary and secondary prevention programs

One of the significant findings of the present study is that there is no intervention addressing primary and secondary prevention for nomophobia. Since this is a new phenomenon and the field is still evolving, intervention studies have focused mainly on designing and testing their efficacy. Limited attempts were made to prevent the development of nomophobia or identify it early and manage its escalation. Evidence from this review suggests that nomophobia interventions focus mainly on symptom reduction rather than preventive models. All studies used participants with high and moderate levels of nomophobia and provided strategies for reducing the existing symptoms. However, there is also a need for preventive interventions, focusing on equipping individuals with skills and digital hygiene strategies. Studies related to mobile phone addictions have shown that early-stage prevention is efficacious and feasible [73–75].

### Theoretical orientations

Theoretical frameworks offer an explanation and variables that can be operationalised while designing the interventions. Theories are fundamental in designing comprehensive interventions [76]. Most studies in this review defined the theoretical foundations used in the interventions. Interventions with strong theoretical foundations contribute to their rigour. It even enables replication of studies and generalising findings to a larger population. Using the CBM framework, it was noticed that there were an equal number of interventions focusing purely on cognition, emotion and eclectic approaches, while just one intervention looked at behaviour. The eclectic interventions, however, had components of behaviour. This indicates a significant imbalance with research being focused more on internal factors like emotion and cognition, rather than maladaptive behaviours that the individual engages in concerning mobile phones.

Additionally, all eclectic approaches had components of behaviour, indicating that when research aims to be more robust and multi-componential, there is a recognition of the necessity to add elements of behaviour along with emotions and cognitions to target the problem at hand holistically. The studies discussed in the review mainly focus on the mindfulness-based or multi-component framework related to cognitive behavioural therapy, like guided imagery, ADL scheduling, sleep hygiene, etc. The cognitive interventions focused on educating individuals about nomophobia and ways to limit phone usage using discussions and video lectures.

Most studies used in this review have conceptualised nomophobia as the anxiety or distress arising from being separated from one’s phone or internet connectivity. The commonly identified predictors mentioned in these studies included FoMO, anxiety, stress, self-esteem, excessive smartphone use and attachment-related distress. Mindfulness based interventions specifically target the emotional predictors of nomophobia like anxiety, FoMO, stress, emotion regulation difficulties and rumination which are all key predictors of nomophobia. Maladaptive cognitions, metacognitive problems, obsessive beliefs, are all cognitive-based predictors of nomophobia were targeted through psychoeducation based interventions and compulsive actions through behavioural interventions. The eclectic interventions had the advantage of being able to target multiple empirically defined risk factors of nomophobia simultaneously. These dimensions and predictors provide a more precise understanding of how different intervention components engage with core features of nomophobia.

### Methodology and outcome focus of the studies

Most studies in this review used the nomophobia questionnaire [7] to assess nomophobia, indicating its wide popularity and applicability. Evidence indicates that maintaining standardised tools to assess intervention outcomes helps improve comparability across studies and the reliability of findings [77]. The Nomophobia questionnaire is a 20-item 7-point Likert scale that assesses all symptoms of nomophobia. However, to increase cultural adaptability, it might be essential to develop more culturally specific instruments. The Indian scale for assessment of nomophobia [44] & The Firat Nomophobia scale [78] are examples of psychometric scales developed in India and Turkey, respectively, to help increase cultural validity. The use of culturally valid scales as pre- and post-measures may better inform the effectiveness of the intervention.

Most studies in this review used the nomophobia questionnaire [7] to assess nomophobia, indicating its wide popularity and applicability. Evidence states that maintaining standardised tools to assess intervention outcomes helps to ease comparability across studies and improve the reliability of findings [77]. The Nomophobia questionnaire is a 20-item 7-point Likert scale that assesses all symptoms of nomophobia.

The design of the studies followed sound randomised controlled trials with a minimum of 7 days and a maximum of 4 months duration. Interestingly, only one study had a follow-up review [56]. Without follow-up data, whether the observed results were sustained over time remains unclear. A noticeable absence of longitudinal studies and interventions spanning longer durations raises questions on long-term efficacy and the practical application of interventions, which has also been pointed out in studies [79].

All the studies in this review focused on quantitative designs and had no additional qualitative component. Insights on how the participants perceived and engaged with the intervention over time, as well as their perceptions on facilitative or deterrent factors, are essential to understand to design efficient and sustainable interventions [80–81]. To better understand the effects of an intervention, it is necessary to employ qualitative and quantitative designs. The limitedness of qualitative studies creates a vacuum for future researchers to design effective solutions. Another method could be objective metrics like measuring screen time, app usage, and subjective questionnaires [71]. These would be objective metrics and thus would be free from any bias. Some interventions in this study did not solely focus on nomophobia. They provided a comprehensive solution for multifaceted issues related to mobile phone dependence and addiction, such as mobile phone addiction, digital dependency, social media addiction, and nomophobia [57]. This diffusion makes isolating efficacy related to nomophobia challenging, thereby limiting the precision of conclusions drawn from these interventions.

### Results, outcomes and limitations of the studies

The review identifies emotion interventions, specifically mindfulness-based ones, were the most common in reducing symptoms of nomophobia. Mindfulness-based interventions help individuals to regulate their emotions more healthily. One of the studies [50] examined a spiritual mindfulness-based intervention and showed how mindfulness leads to lower stress and cortisol production. Lowering cortisol leads to reduced anxiety associated with mobile phones. It also teaches individuals self-acceptance, enabling acceptance of thoughts that arise in the absence of phones rather than resisting or fearing them. Literature has suggested that mindfulness has a significant impact on alleviating levels of anxiety [82], which might be the reason it is being adopted as a therapy for mobile phone-related anxiety as well. The effectiveness of the emotion and mindfulness-based interventions can also be reviewed from the lens of the attachment theory. Nomophobia or the anxiety resulting from separation from one’s smartphone, could be categorised as attachment-related anxiety. Mindfulness-based interventions can help promote awareness in the present moment, improve self-regulation and distress tolerance, reducing the anxiety arising when individuals are disconnected from their devices.

Although not as widely used as mindfulness-based interventions, technology-based and cognitive interventions were also used. The technology-based interventions were not as effective in dealing with nomophobia, which could be owing to the paradox of using technology to deal with an issue stemming from technology itself. The cognitive psychoeducation intervention was less common but effective in dealing with nomophobia. By assisting individuals to recognise that they may be susceptible to nomophobia these interventions spread awareness and lay the foundation for early acknowledgement and behaviour change. The effectiveness of the psychoeducation or cognitive interventions and the behavioural interventions can be understood through the theoretical framework of the CBM. These interventions focus on changing maladaptive thoughts and irrational beliefs by educating individuals on the nature and symptoms of nomophobia. Educating them on unfamiliar topics and promoting healthy digital usage therefore was the first step towards expected change in behaviour.

The multi-component interventions, which use an eclectic approach, were effective but had varying components, making it difficult to isolate the specific aspects contributing to the results. Some had mindfulness components, showing their efficacy in mitigating this condition. The sports intervention was effective, likely due to its role as a distraction from technology, its biological impact on reducing stress, and its ability to promote social interaction. In the behaviour intervention, the client-centred approach also proved to lower levels of nomophobia due to its ability to show unconditional positive regard and empathy to the client, thus decreasing their levels of anxiety. Studies have also demonstrated the efficacy of client-centred therapy for anxiety [83–84]. Eclectic interventions have an advantage of combining multiple theoretical orientations to target nomophobia which could ensure that positive effects would sustain over a longer period. They work on mindfulness through mood tracking, physical symptoms as well as negative thoughts to ensure all domains are given equal importance.

In addition to the noteworthy findings, these studies also discussed a few limitations. Many studies spoke about how generalisability is limited. Most studies had homogeneous participants. Despite cultural diversity across the studies, each study focused on one geographic location, limiting generalisability. Few studies also had a small number of participants; for example, one study by Maghfiroh et al. [55] had only 10 participants. Another standard limitation revealed by most studies was that all questionnaires were self-reported, raising concerns about response bias and social desirability.

These methodological restrictions in the research studies reviewed may affect the reliability of the results and mask the interventions’ true success. Future studies on nomophobia interventions will benefit from being implemented across diverse cultural samples and incorporating mixed methods to enhance the sustainability of their efficacy.

## Strengths, limitations and future recommendations of this review

The primary strengths of this review reside in its focus on a topical issue that requires prompt assessment and action. The methodological framework was strictly adhered to and transparently outlined in the article [46]. The authors employed Zotero [85], a bibliographic manager, to systematically organise all references and citations. Furthermore, they utilised seven databases to expand the review’s scope, thus gathering as much evidence as possible. Since this is a relatively new topic and not widely recognised, the search terms and keywords were kept broad to ensure greater depth and accuracy in the search results. There is considerable variability in the selected articles regarding cultural diversity. These articles come from different countries, which helps provide a broader range of results.

However, it is equally important to recognise the limitations it presents. The article only included publications in English, which may have resulted in the omission of significant works published in other languages, specifically regionally and culturally adapted scales and interventions. Grey literature was also not included in the review. Additionally, this study summarises the features of the interventions but does not rank them in terms of their effectiveness.

Future research recommendations can be made in this area based on the findings of this review. Interventions could be specifically designed to tackle nomophobia rather than combining it with multiple concerns. These interventions should be rooted in psychological theories, enhancing the understanding of their successes or failures and providing evidence for further interventions. Studies need to develop preventive intervention models that address at-risk populations, rather than relying solely on curative approaches that respond only after the issue has escalated. There is a need for larger, multi-site studies that include participants from diverse cultural, educational, and socioeconomic backgrounds to better assess the generalisability and effectiveness of the intervention. Long-term follow-up over several months is essential to evaluate the sustainability of the observed changes and promote generalisation.

## Implications

The study implies the need for more interventions to deal with nomophobia. The findings of this review emphasise how technological overreliance is not just a personal issue but a public mental health concern. As nomophobia becomes more prevalent, especially in younger populations, there is a need to foster societal awareness and reduce the stigma around seeking help for digital dependency. At the individual level, nomophobia affects emotional regulation, stress, anxiety, and self-perception. The review indicates that effective interventions can improve a person’s self-awareness and coping strategies, promote healthier digital habits, and improve overall mental health.

The review highlights the need for large-scale interventions to be applied. Schools and universities are ideal platforms for implementing preventive and early intervention strategies. Psychoeducation programs, digital well-being curricula, and group-based interventions can help students develop healthier digital habits and improve focus, which may positively impact academic performance. Nomophobia is not limited to one culture or country; the studies reviewed spanned various geographical regions, showing that this issue is global in scope. Addressing nomophobia can contribute to digital equity, healthier digital ecosystems, and cross-cultural mental health promotion.

## Conclusion and recommendations

This study summarises the content, frequency, outcomes, duration, and efficacy of the available interventions targeting nomophobia. Based on the PRISMA-ScR, a scoping literature review of 12 studies was conducted. Four major types of interventions were included: Mindfulness-based, Psychoeducation, Technology-regulated and Multi-component interventions. The interventions were conducted online, in-person, and hybrid, mainly with the young adult population. However, the domain is still in its nascent stage, with most of the studies combining nomophobia interventions with other mobile phone addiction and social media addiction interventions. As a result, the specific impact of interventions on nomophobia alone remains difficult to isolate. There is a clear gap in the literature regarding interventions designed exclusively for nomophobia, and this is an area future researchers are strongly encouraged to explore.

Interventions developed should target a wider variety of populations, not just limiting themselves to young adult groups, since mobile phones have now become a part of everyone’s lives. School- and college-based programs can serve as efficient platforms for early prevention and outreach to wider populations in a structured and scalable way. This scoping review highlights the need for high-quality, methodologically rigorous interventions to help reduce levels of nomophobia among different population groups. Additionally, long-term follow-up data and the incorporation of qualitative data were lacking in most studies, limiting our understanding of sustained impact and the mechanism of change.

## Data Availability

No datasets were generated or analysed during the current study.
